# Synthesis of C-Plane Oriented Hexagonal Tungsten Oxide Membranes on Tubular Substrates and Their Acetic Acid/Water Separation Performances

**DOI:** 10.3390/membranes11010038

**Published:** 2021-01-05

**Authors:** Hiroto Kunishi, Shintaro Wada, Yuki Kamimoto, Ryoichi Ichino, Yan Lin, Long Kong, Liang Li, Takeshi Hagio

**Affiliations:** 1 Department of Chemical Systems Engineering, Graduate School of Engineering, Nagoya University, Furo-cho, Chikusa-ku, Nagoya, Aichi 464-8603, Japan; kunishi.hiroto@d.mbox.nagoya-u.ac.jp (H.K.); yuki.kamimoto@mirai.nagoya-u.ac.jp (Y.K.); ichino.ryoichi@material.nagoya-u.ac.jp (R.I.); 2 Department of Materials Science and Engineering, School of Engineering, Nagoya University, Furo-cho, Chikusa-ku, Nagoya, Aichi 464-8603, Japan; wada.shintaro@d.mbox.nagoya-u.ac.jp; 3 Institute of Materials Innovation, Institutes of Innovation for Future Society, Nagoya University, Furo-cho, Chikusa-ku, Nagoya, Aichi 464-8601, Japan; 4 School of Environmental Science and Engineering, Shanghai Jiao Tong University, 800 Dongchuan Road, Shanghai 200240, China; linyan2002@sjtu.edu.cn (Y.L.); longmao88@sjtu.edu.cn (L.K.); liangli117@sjtu.edu.cn (L.L.)

**Keywords:** tubular substrate, c-plane orientation, hexagonal tungsten oxide membranes, seeding, dehydration, acetic acid/water mixtures

## Abstract

Hexagonal tungsten oxide (h-WO_3_) membrane is a novel candidate for dehydration of acetic acid (CH_3_COOH)/water mixtures owing to its molecular sieving property and acidic resistance. Meanwhile, c-plane orientation is an important factor for h-WO_3_ membranes because the pores of h-WO_3_ run along its c-axis. However, so far, high c-plane orientation has not been successful on tubular substrates. Here, the effect of synthesis conditions of h-WO_3_ membranes on tubular substrates against c-plane orientation and CH_3_COOH/water separation performance are investigated. The h-WO_3_ membranes were prepared by hydrothermal synthesis from a precursor sol containing various amounts of sodium tungstate (Na_2_WO_4_) in the presence of tubular substrates with seeds embedded on their outside surface. The seeding method and the amount of Na_2_WO_4_ in the precursor sol significantly affected both crystal orientation and densification of the membrane. A precursor sol with appropriate amounts of Na_2_WO_4_ was essential to simultaneously satisfy high c-plane orientation and densification of the membrane while excess Na_2_WO_4_ drastically decreased the degree of c-plane orientation. A highly c-plane oriented h-WO_3_ membrane was successfully obtained under the optimized condition, which exhibited a maximum separation factor of 40.0 and a water permeance of 1.53 × 10^−7^ mol·m^−2^·s^−1^·Pa^−1^ in a 90:10 wt % CH_3_COOH/water mixture. The water permeance approximately doubled compared to the previous report, possibly owing to the significantly higher degree of c-plane orientation. Furthermore, it was found that its separation ability can be maintained while stored in 90:10 wt % CH_3_COOH/water mixture with pH < 0 for more than 500 h.

## 1. Introduction

Dehydration of organic solvents is one of the most important technologies in chemical processes. Currently, the primary technology for dehydration of organic solvents is distillation; however, distillation-based separations are energy consuming for azeotropic and close-boiling-point mixtures [1]. Therefore, an energy-efficient separation technology is desired to reduce the environmental load of these processes. Membrane-based separations are expected to be energy-efficient technologies for dehydration of azeotropic and close-boiling-point mixtures because their separations are driven by the differences in molecular size, resulting in different diffusion rates across the membrane, instead of their difference in boiling points [2]. Indeed, a hybrid process using both membranes and distillation towers or a single process by using only membranes can significantly reduce the energy consumption for dehydration of organic solvents when compared to using only distillation towers [3,4,5,6]. Thus, separation membranes suitable for dehydration of organic solvents are important to realize an environmental-friendly, sustainable process.

Among numerous organic solvents, acetic acid (CH_3_COOH) is one of the most important basic chemical material in chemical industries with a large production amount [7,8]. It has high demand for dehydration because separation of water from CH_3_COOH is an important process involved in the production of vinyl acetate, vitamins, terephthalic acid, cellulose esters and other chemicals [9]. A huge amount of energy is necessary for dehydration of CH_3_COOH by distillation because there is only small difference in volatility between CH_3_COOH and water in concentrated CH_3_COOH solutions, requiring large reflux flow rate [3,8,9]. Membrane pervaporation (PV) has attracted widespread attention for dehydrating CH_3_COOH/water mixtures owing to its easy control and high efficiency [10]. Inorganic membranes, especially zeolite membranes consist of MOR-type [10,11,12], MFI-type [13,14,15], MER-type [16], CHA-type [17,18] and DDR-type zeolites [9,19,20], have exhibited excellent PV performances against CH_3_COOH/water mixtures; however, the strict requirements in acidic stability still limits their application. Thus, new membrane materials with high acidic stability are of interest.

Hexagonal tungsten oxide (h-WO_3_) is an interesting membrane material for dehydration of CH_3_COOH/water mixtures because it possesses pores of molecular dimension running along its c-axis [21] and high stability in acids with pH lower than 4 [22]. The pores have been reported to have openings of 0.367 nm [23], which is larger than the diameter of the water molecule (0.296 nm) and smaller than the diameter of the CH_3_COOH molecule (0.436 nm) [24], suggesting separation by molecular sieving. Therefore, if we can prepare dense h-WO_3_ membranes with the pores running across the membrane; i.e., dense c-plane oriented h-WO_3_ membranes, it shall be promising to apply for the dehydration of CH_3_COOH/water mixtures. However, the proposed c-plane oriented structure is thought to be difficult to form since h-WO_3_ crystals generally crystallizes in a whisker-like structure with extremely small c-plane area [25].

Recently, our group discovered that hydrothermal synthesis in the presence of CH_3_COOH promotes c-plane growth of h-WO_3_ and enables the formation of a dense h-WO_3_ membrane on porous substrates [26]. However, although the preparation conditions were optimized before using flat-type substrates, the high degree of c-plane orientation could not be replicated when using tubular substrates, which are more attractive for industrial applications. Namely, the c-plane orientation drastically decreased for tubular substrates even when applying the condition that derived almost complete c-plane orientation on the flat-type substrate (See Figure S1 in Supplementary Material). This indicates that the preparation conditions on tubular substrates must be adjusted apart from flat-type substrates. Here, we attempt to prepare a dense and highly c-plane oriented h-WO_3_ membrane on a tubular substrate by modification of both seeding method and precursor sol composition apart from our previous study. Two different seeding methods and four different precursor sol compositions were considered. The CH_3_COOH/water separation performance of the prepared membranes were evaluated by means of PV experiments. In addition, its acidic resistance and performance stability was checked through a long-term PV experiment in a 90:10 wt % CH_3_COOH/water mixture.

## 2. Materials and Methods

### 2.1. Materials

Asymmetric porous α-alumina tubes with an outer diameter of 12 mm, wall thickness of 1.5 mm, length of 500 mm, overall porosity of ~35% and outer surface pore size of 0.1 μm were purchased from NGK Filtech Co., Ltd., Chigasaki, Japan The former tubes were cut into pieces with lengths of 10 mm using a cutting machine and were adopted as the tubular substrate for the membranes. The tubular substrates were completely dried at 343 K after removing contaminants on the surface by carrying out sonication in distilled water and ethanol (purity: 95%) for 10 min, respectively. Sodium tungstate dihydrate (Na_2_WO_4_∙2H_2_O, purity: >98%), CH_3_COOH (purity: >99%) and distilled water were used for membrane synthesis. CH_3_COOH and distilled water were also used in the PV experiments. All chemical reagents used for membrane synthesis and performance tests were purchased from Nacalai Tesque Inc., Kyoto, Japan and were used as-received. Distilled water was produced in our laboratory using a water distillation unit (RFD240NC, Toyo Seisakusho Kaisha, Ltd., Kashiwa, Japan).

### 2.2. Preparation of Seeds and Seeding on Tubular Substrates

**Figure 1:**
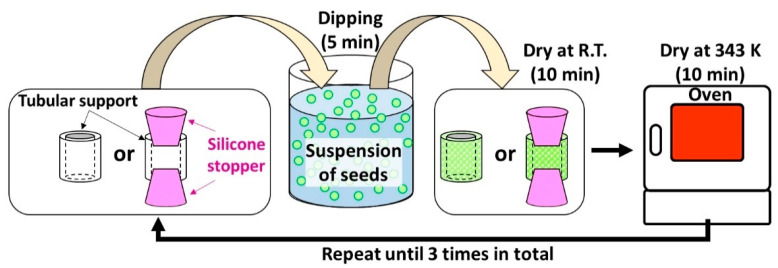
Illustration of two seeding methods: seeding in absence or presence of silicone stoppers.

In total, 36.75 g of CH_3_COOH was rapidly poured into an Na_2_WO_4_ solution of 27.30 g containing 7.0 mmol of Na_2_WO_4_, under continuous agitation. Precipitates appeared immediately after CH_3_COOH addition, forming a suspension of seeds. Next, seeding was performed by dipping the tubular substrates into the aforementioned suspension for 5 min and subsequently dried at room temperature and 343 K for 10 min, respectively. This seeding process was repeated three times to ensure attachment of seeds on the surface of tubular substrates. In this study, two ways of seeding were tested. One is directly dipping the tubular substrates into the seed suspension as we have done in our previous study. Another is dipped into the seed suspension with both ends of the tubular substrates sealed using silicon stoppers to avoid attachment of seeds on the inner wall of the tubular substrates. The two seeding methods are illustrated in Figure 1.

### 2.3. Membrane Synthesis

The membranes were synthesized on the outer surface of the tubular substrates. Membrane synthesis was carried out using precursor sols prepared by mixing CH_3_COOH with Na_2_WO_4_ solutions with a variety of concentrations as follows. Na_2_WO_4_ solutions were made by completely dissolving Na_2_WO_4_∙2H_2_O in distilled water. To this solution, CH_3_COOH was added dropwise under continuous agitation until the pH reached 2 ± 0.2 and was continuously mixed for an additional 5 min. The resulting precursor sols were approximately 60 mL and the amount of Na_2_WO_4_ in the sols were 3.5, 5.0, 6.0 or 7.0 mmol, respectively. Then, the seeded tubular substrates prepared in Section 2.2 were fixed on a handmade polytetrafluoroethylene holder and placed in polytetrafluoroethylene vessels. The aforementioned precursor sol was slowly poured into the vessels. The polytetrafluoroethylene vessels were sealed in a stainless-steel autoclave (HU-100, SAN-AI Kagaku Co. Ltd., Nagoya, Japan) and transferred to a convection oven (MOV-450, AS ONE Corporation, Osaka, Japan) in order to perform hydrothermal synthesis at 453 K for 24 h. After the oven was cooled down to room temperature, the membranes were rinsed by sonicated in distilled water and ethanol several times to clean the membrane surface and to remove the precursor sol remaining inside the tubular substrate. Finally, the membranes were dried at 343 K overnight and were stored at room temperature.

### 2.4. Characterization of Seeds and Membranes

The crystalline phases of the seeds and membranes were characterized using X-ray diffraction (XRD, Ultima IV, Rigaku Corporation, Akishima, Japan) equipped with Cu-Kα radiation (λ = 1.54050 Å). The degrees of c-plane orientation of the membranes were evaluated using the peak intensities of (001) plane and (200) plane of h-WO_3_, which are the representative c-plane and a-plane, respectively. To quantitatively compare the degree of c-plane orientation, c-plane orientation index; *R*, defined by the following Equation (1) was adopted.

*R* = (*I*_m_001_/*I*_m_200_)/(*I*_p_001_/*I*_p_200_) (1)

Here, *I*_m_001_ and *I*_m_200_ are the peak intensities of (001) and (200) planes of h-WO_3_ obtained from the membranes and *I*_p_001_ and *I*_p_200_ are the peak intensities of (001) and (200) plane registered in the standard powder diffractogram (ICDD #01-075-2187). *R* > 1 indicates that the membrane exhibits c-plane orientation and a larger *R* corresponds to a higher degree of c-plane orientation. Meanwhile, *R* = 1 or *R* < 1 indicate that the membranes exhibit random (equivalent to standard powder data) or a-plane orientation, respectively. The morphologies of the seed, surfaces and cross-sections of the membranes were observed using a scanning electron microscope (SEM, S-4800, Hitachi High-Tech Corporation, Tokyo, Japan).

### 2.5. Evaluation of Membrane Performance

The PV performances of the membranes were considered from the separation performance against 90:10 wt % CH_3_COOH/water mixtures. The membrane was soaked in the 90:10 wt % CH_3_COOH/water mixtures and the testing temperature and time were fixed to be 353 K and 10 h. The permeate collected within the first hour was discarded to make sure the performance stabilized and the permeate thereafter was collected for analysis. The change in feed concentration by discarding the permeate collected in the first hour was less than 0.005%. Separation factor; α, was calculated using following Equation (2):

α= (*X*_1_/*Y*_1_)/(*X*_0_/*Y*_0_) (2)

Here, *X*_0_ and *X*_1_ are the mass fractions of water in the feed and in the permeate and *Y*_0_ and *Y*_1_ are the mass fraction of CH_3_COOH in feed and in the permeate, respectively.

Additionally, the total flux and permeability of each component was evaluated. The total flux was the weight of the permeate per unit membrane area collected in one hour. The permeability of a particular component (*P*_c_) was calculated using the following Equation (3):

*P*_c_ = ((*m* × *x*_c_)/M_c_)/(*A* × (*t*⁄3600) × Δ*p*_c_) (3)

Here, *m* is the amount of the collected permeate (g), *x*_c_ is the mass fraction of the target component (g/g), M_c_ is the molecular weight of the target component and Δ*p*_c_ is the partial pressure differences (Pa) of the target component across the membrane, respectively. The CH_3_COOH concentrations of the feed and the permeate were measured using a reflectometer (PAL-RI, Atago Co. Ltd., Tokyo, Japan).

## 3. Results

### 3.1. Characterization of the Seed

**Figure 2:**
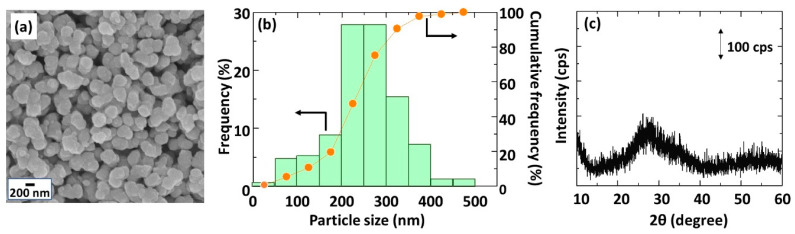
Characterization of seeds used in this study: (**a**) SEM image; (**b**) Particle size distribution; (**c**) XRD diffractogram.

The seed size against the pore size of the porous substrate is reported to be an important parameter in membrane synthesis. If the seed is too small compared to the substrate pores, the seed will penetrate into the substrate and will not remain on its surface [27]. In contrast, if it is too large, the seed layer will become inhomogeneous and defective [28]. The appropriate seed size is considered to be smaller than 1.5 m to obtain a homogeneous surface [27] but larger than the substrate pores. Thus, the seeds and the tubular substrates were characterized. The SEM image, particle size distribution, and XRD diffractogram of the seeds are shown in Figure 2. The morphology of the seed was isotropic irregular shape (Figure 2a) with an average diameter of 253 nm. Approximately 90% of the seed size was between 100 and 500 nm, which is expected to be effective to use as seeds for substrate with a nominal outer surface pore size of 0.1 m (100 nm). The seed had an amorphous structure according to XRD as shown in Figure 2c.

**Figure 3:**
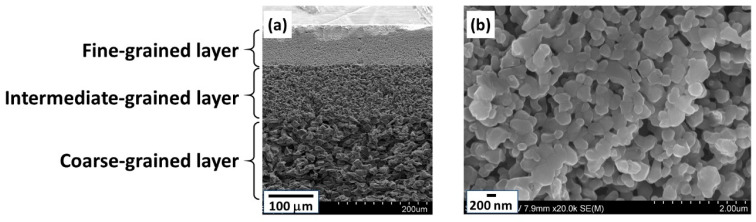
Characterization of the tubular substrate: (**a**) cross-sectional SEM image; (**b**) outer surface (fine-grained layer) SEM image.

Cross-sectional and surface SEM images of the tubular substrates are shown in Figure 3. The tubular substrates consist of three different layers; fine-grained, intermediate-grained and coarse-grained layers, as shown in Figure 3a. The fine-grained layer comprising the outer surface of the tubular substrates was approximately 100 μm thick and consist of well packed alumina particles of several hundred nanometers as shown in Figure 3b. The spaces between the particles were mostly smaller than the seeds and no large defects could be found. This observation also supports that the relationships between seed size and substrate pores were adequate for effective seeding.

### 3.2. Effect of Seeding on Tubular Substrates

**Figure 4:**
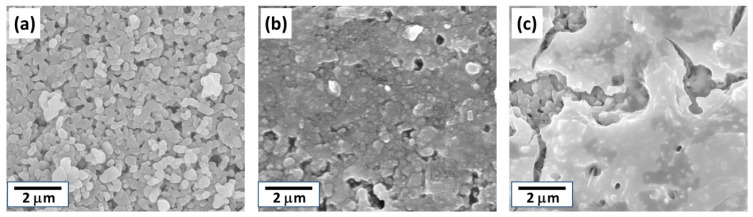
Surface SEM images of the tubular substrate: (**a**) before seeding; (**b**) after seeding in the absence of silicon stoppers; (**c**) after seeding in the presence of silicon stoppers.

**Figure 5:**
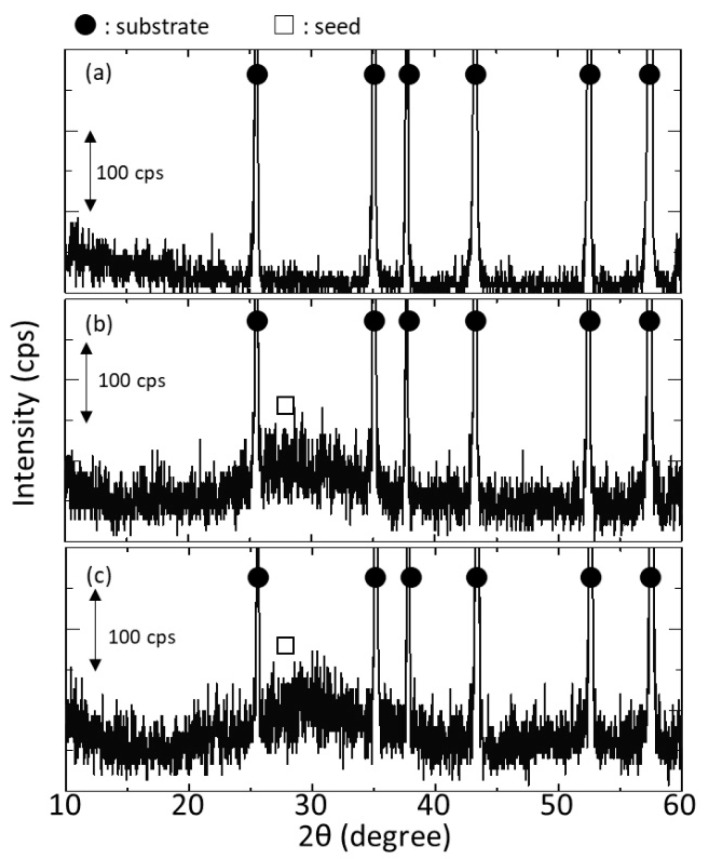
XRD diffractograms of the tubular substrates: (**a**) without seeding; (**b**) with seeding in the absence of silicon stoppers; (**c**) with seeding in the presence of silicon stoppers.

Figure 4 shows the surface SEM images of the tubular substrate before and after seeding with and without the silicon stoppers at both ends of the tubular substrate. Seeds almost cover the surface after seeding in both cases; however, the attached seed layer seemed to be thicker when using silicon stoppers. For the sample seeded without silicon stoppers, some areas showed morphology similar to the substrate surface, implying that the seed layer was relatively thin. Meanwhile, for the sample with silicon stoppers, although some cracks were observed, the surface morphology completely changed possibly due to a thicker seed layer formation. Figure 5a–c show the XRD diffractogram of the tubular substrate before seeding and after seeding without and with silicon stoppers. After seeding, a broad peak was detected between 25 to 35 degrees in both cases. This is expected to be derived from the amorphous h-WO_3_ seed layers, considering the XRD diffractogram of the seed shown in Figure 2c. The intensity of the broad peak was slightly stronger for the sample seeded in the presence of silicon stoppers, implying thicker seed layer formation as anticipated from the SEM image. This might be owing to the air remaining inside the tube when both ends are sealed with silicon stoppers, leading to suction and filtration of the seed solution into the substrate.

**Figure 6:**
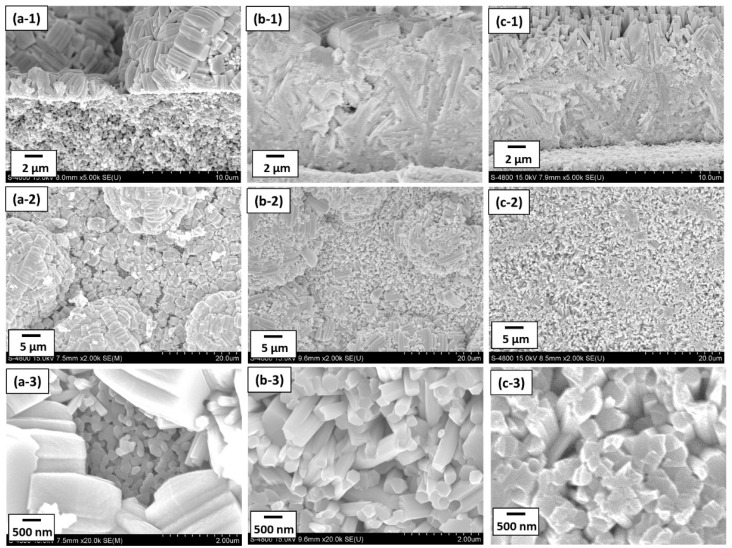
Cross-sectional and surface SEM images of the h-WO_3_ membranes synthesized at 453 K for 24 h: (**a-1**) cross-sectional SEM image of the membrane prepared without seeding; (**a-2**) low and (**a-3**) high magnification surface SEM images of the membrane prepared without seeding; (**b-1**) cross-sectional SEM image of the membrane prepared with seeding in the absence of silicon stoppers; (**b-2**) low and (**b-3**) high magnification surface SEM images of the membrane prepared with seeding in the absence of silicon stoppers; (**c-1**) cross-sectional SEM image of the membrane prepared with seeding in the presence of silicon stoppers; (**c-2**) low and (**c-3**) high magnification surface SEM images of the membrane prepared with seeding in the presence of silicon stoppers.

**Figure 7:**
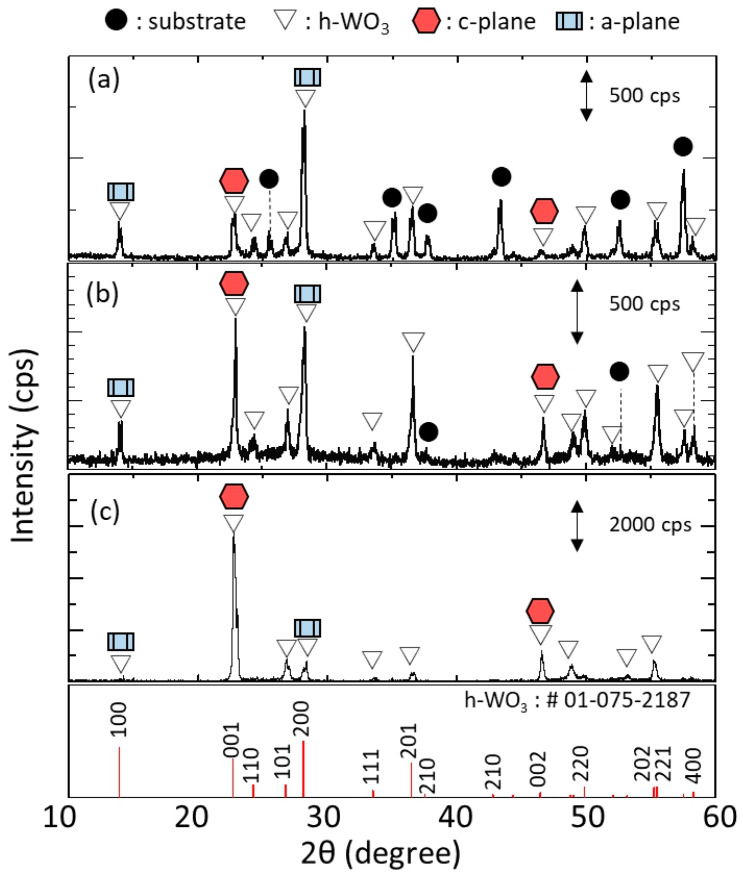
XRD diffractograms of the membranes synthesized at 453 K for 24 h: (**a**) without seeding; (**b**) with seeding in the absence of silicon stoppers; (**c**) with seeding in the presence of silicon stoppers.

The effect of seeding was further investigated by synthesizing the h-WO_3_ membranes without and with seeding using precursor sols containing 3.5 mmol of Na_2_WO_4_. Figure 6 and Figure 7 show the SEM images and the XRD diffractograms of the membranes synthesized at 453 K for 24 h without and with seeding. When using the tubular substrates without seeding, although rod-like crystals were deposited on the substrate surface, the thickness of membrane was uneven (Figure 6a-1) and the substrate surface was not completely covered (Figure 6a-2). The substrate was clearly visible in the gaps between the crystals at high magnification as shown in Figure 6a-3, indicating that the densification of the membrane was insufficient. Moreover, the rod-like crystals seem to be mainly tilted parallel to the substrate surface, implying a rather a-plane orientation of crystals. This was further confirmed from its XRD diffractogram showing a highest peak from reflection of a-plane of h-WO_3_ as shown in Figure 7a. This may be due to the less nucleation occurring on the substrate surface in the absence of seeds. Details will be discussed afterwards comparing the results with seeding.

**Figure 8:**
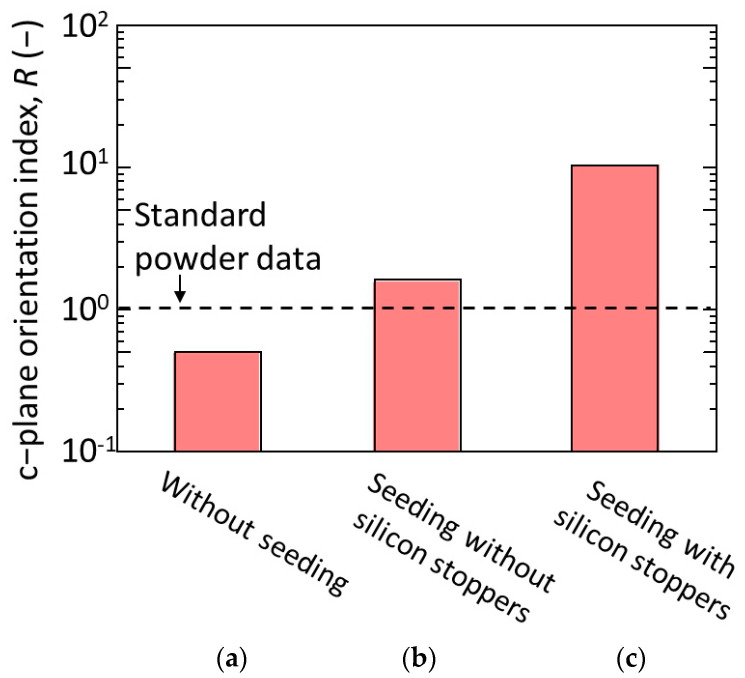
C-plane orientation index of membranes synthesized at 453 K for 24 h: (**a**) without seeding; (**b**) with seeding in the absence of silicon stoppers; (**c**) with seeding in the presence of silicon stoppers.

Meanwhile, when using the tubular substrates with seeding, dense membranes with thickness of more than 10 μm were formed and rod-like crystals seemed to well cover the surface of the substrate regardless of the use of silicon stoppers, as shown in Figure 6b,c, respectively. This indicates that the seeding was effective to increase the homogeneity of the membrane and promote their densification. In contrast, the appearances of the membrane surfaces were quite different when using substrates seeded without and with the silicon stoppers. The rod-like crystals stood more vertically to the substrate surface when membranes were synthesized using tubular substrates seeded in the presence of silicon stoppers. According the XRD diffractograms of the synthesized membranes, the reflection from (200) plane; a-plane of h-WO_3_ drastically decreased while the reflection from (001) plane; c-plane of h-WO_3_ drastically increased when employing tubular substrates seeded using silicon stoppers (Figure 7c). This means that the seeding method significantly affects the crystal orientation of the h-WO_3_ membranes. Figure 8 shows the c-plane orientation index; *R* of the membranes synthesized using substrates without and with seeding in the absence or presence of silicon stoppers. Seeding without silicon stoppers was found to slightly improve the c-plane orientation of the membrane; however, the c-plane orientation index is still similar to the value of the standard powder diffraction, meaning that c-plane orientation was still weak. In contrast, the c-plane orientation index increased approximately ten times when substrates were seeded with silicon stoppers, clearly evidencing the effectiveness of sealing both ends of the tubular substrate with the silicon stoppers. This improvement may be owing to the thicker seed layer formed on the substrate surface.

**Figure 9:**
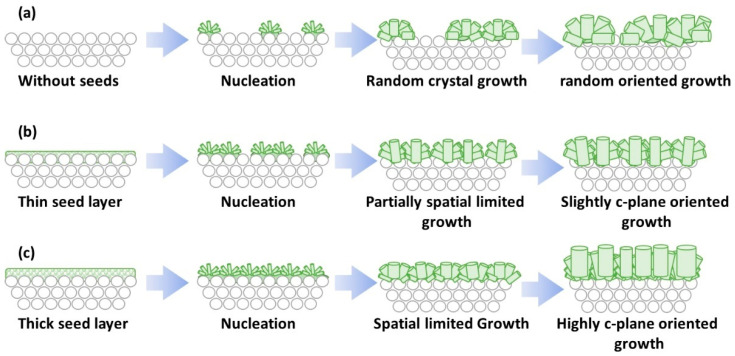
Illustration of the anticipated mechanism of the different crystal orientation observed for h-WO_3_ membranes prepared under different seeding conditions: (**a**) without seeding; (**b**) with seeding in the absence of silicon stoppers; (**c**) with seeding in the presence of silicon stoppers.

The mechanism of how the seeding and its amount effected the crystal orientation of the membrane may be explained as follows. When no seeds existed on the surface of the tubular substrate, the number of nuclei that nucleated on the substrate surface during the initial stage of hydrothermal synthesis should be small. Thus, the open space between each nuclei shall be large, leaving enough area for the nuclei to grow freely. This must have allowed crystal growth of h-WO_3_ crystals in random directions as shown in Figure 9a. When a thin seed layer exists, the nucleation should be slightly promoted and the number of nuclei crystallized on the substrate surface shall increase. Thus, the space between the nuclei shall become smaller and the growth of the h-WO_3_ pillars shall be disturbed by the neighboring crystals. Therefore, spatially limited growth must have partially took place, as shown in Figure 9b, resulting in a slight c-plane orientation. Furthermore, in the case of thicker seed layer, h-WO_3_ may be more likely to crystallize near the substrate surface during hydrothermal synthesis. The growth of the h-WO_3_ crystals comprising the membrane is almost limited in two dimensions due to the substrate, and neighboring crystals spatially limit the growth of rod-like crystals parallel to the substrate surface. Therefore, the crystals may have grown vertically to the substrate surface as shown in Figure 9c. In addition, because the seeds did not attach on the inner walls of the tubular substrate, the tungsten source in the precursor sol may have been selectively consumed at the outer surface of the tubular substrate, leading to increase in crystallization of neighboring crystals. This must have led to membrane formation with c-axes oriented vertical to the surface of the underlying substrate. Silicon stopper was applied hereafter to ensure efficient seeding, to prevent seeding on the inner wall of the tube, and to promote the c-plane orientation of the final membrane.

### 3.3. Effect of Na_2_WO_4_ Concentration in Precursor Sol on the Orientation of h-WO_3_ Membrane

**Figure 10:**
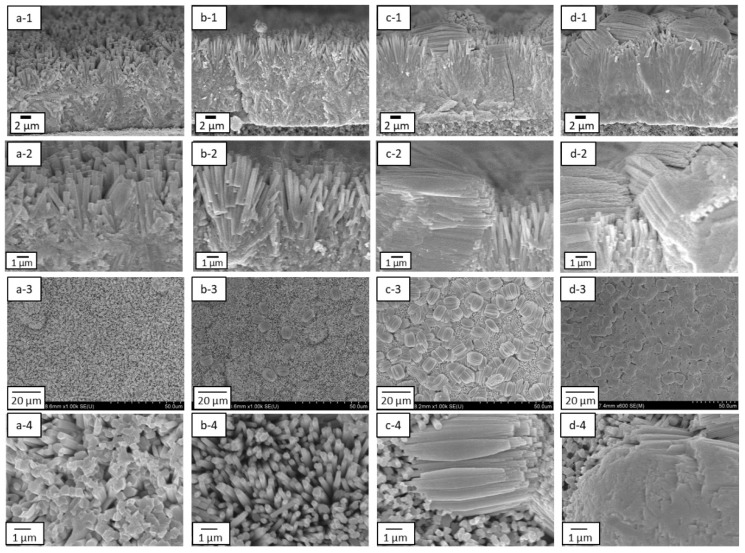
Cross-sectional and surface SEM images of the membranes synthesized using precursor sols containing different amounts of Na_2_WO_4_: (**a-1**) low and (**a-2**) high magnification cross-sectional SEM images of the membrane synthesized using precursor sol containing 3.5 mmol of Na_2_WO_4_; (**a-3**) low and (**a-4**) high magnification surface SEM images of the membrane synthesized using precursor sol containing 3.5 mmol of Na_2_WO_4_; (**b-1**) low and (**b-2**) high magnification cross-sectional SEM images of the membrane synthesized using precursor sol containing 5.0 mmol of Na_2_WO_4_; (**b-3**) low and (**b-4**) high magnification surface SEM images of the membrane synthesized using precursor sol containing 5.0 mmol of Na_2_WO_4_; (**c-1**) low and (**c-2**) high magnification cross-sectional SEM images of the membrane synthesized using precursor sol containing 6.0 mmol of Na_2_WO_4_; (**c-3**) low and (**c-4**) high magnification surface SEM images of the membrane synthesized using precursor sol containing 6.0 mmol of Na_2_WO_4_; (**d-1**) low and (**d-2**) high magnification cross-sectional SEM images of the membrane synthesized using precursor sol containing 7.0 mmol of Na_2_WO_4_; (**d-3**) low and (**d-4**) high magnification surface SEM images of the membrane synthesized using precursor sol containing 7.0 mmol of Na_2_WO_4_.

Figure 10 shows SEM images of the membranes synthesized using precursor sols containing 3.5, 5.0, 6.0 and 7.0 mmol of Na_2_WO_4_, respectively. Dense membranes seemed to form in all conditions. The thicknesses of the formed membranes were found to increase from around 10 μm to approximately 15 μm by increasing the amount of Na_2_WO_4_ in the precursor sol from 3.5 to 7.0 mmol. The amount of Na_2_WO_4_ in the precursor sol was also found to affected the membrane structure. The membranes synthesized with 3.5 and 5.0 mmol of Na_2_WO_4_ were comprised of rod-like crystals oriented vertically to the substrate surface, as shown in Figure 10a-2,b-2, while those synthesized with 6.0 and 7.0 mmol of Na_2_WO_4_ contained many rod-like crystals facing parallel to the substrate surface as shown in Figure 10c-2,d-2.

**Figure 11:**
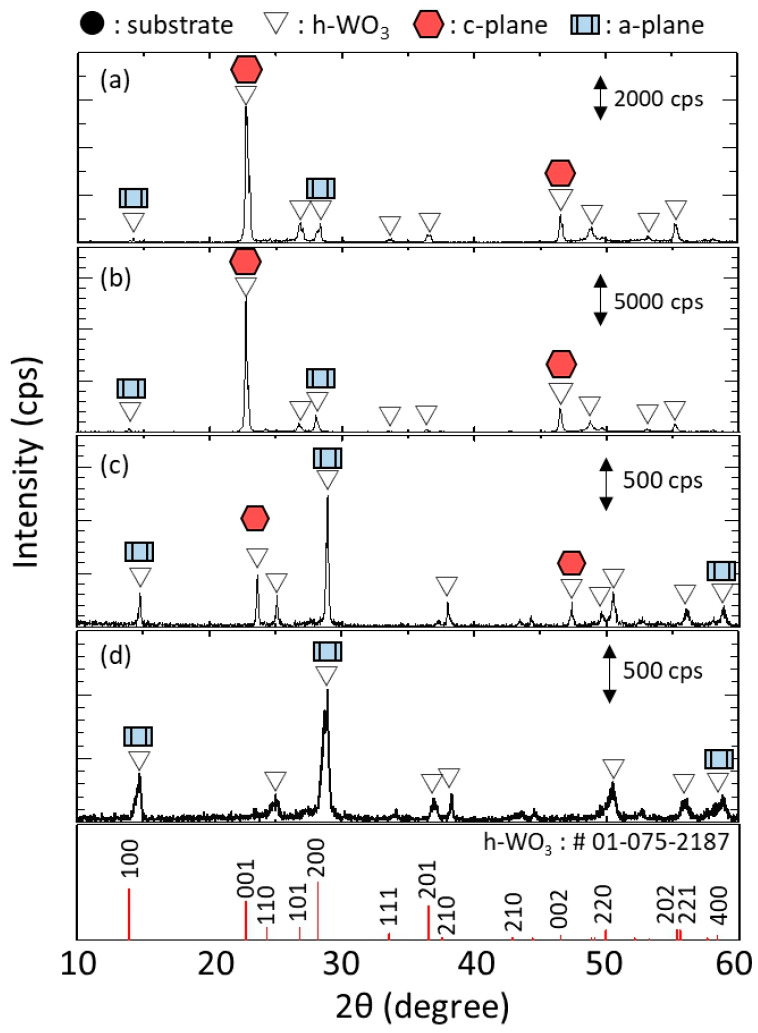
XRD diffractograms of the membranes synthesized using precursor sols containing different amounts of Na_2_WO_4_: (**a**) 3.5 mmol; (**b**) 5.0 mmol; (**c**) 6.0 mmol; and (**d**) 7.0 mmol.

Figure 11 shows the XRD diffractograms of h-WO_3_ membranes synthesized using precursor sols containing 3.5, 5.0, 6.0 and 7.0 mmol of Na_2_WO_4_, respectively. Only peaks related to h-WO_3_ were detected and those from the substrate were not, indicating that the h-WO_3_ membranes without large defects formed thicker than the penetration depth of the X-ray. The peaks derived from c-plane and a-plane of h-WO_3_ are marked with hexagonal symbols and rectangular symbols, respectively. The membranes synthesize with 3.5 and 5.0 mmol of Na_2_WO_4_ showed obviously higher intensity of c-plane than a-plane of h-WO_3_ while those synthesize with 6.0 and 7.0 mmol of Na_2_WO_4_ showed the opposite trend. This matched well with the change in morphology of the membranes observed from the SEM images shown in Figure 10; i.e., change in direction of rod-like crystals from vertical to parallel to the substrate surface. This implies that an excess amount of Na_2_WO_4_ in the precursor sol has an adverse effect on the c-plane orientation of the membranes.

**Figure 12:**
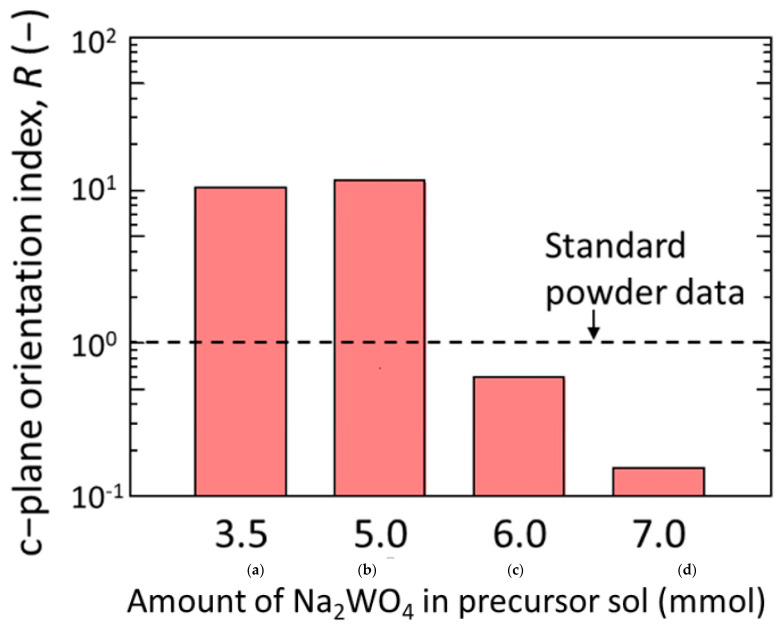
C-plane orientation index of membranes synthesized using precursor sols containing different amount of Na_2_WO_4_: (**a**) 3.5 mmol; (**b**) 5.0 mmol; (**c**) 6.0 mmol; and (**d**) 7.0 mmol.

The effect of Na_2_WO_4_ amount on the degree of c-plane orientation of the h-WO_3_ membranes was quantitatively evaluated by the c-plane orientation index; *R*, as shown in Figure 12. The *R* of the membranes synthesized with both 3.5 and 5.0 mmol of Na_2_WO_4_ showed 10 times higher value than that of the standard powder data, which were 10.4 and 11.3, respectively. This was also significantly higher than that of our previous report; which showed a maximum *R* of 2.0, meaning a membrane with high c-plane orientation has been realized on the tubular substrate. Meanwhile, when the amount of Na_2_WO_4_ in the precursor sol was further increased to 6.0 and 7.0 mmol, *R* drastically decreased to a lower value than 1. Especially, when the amount of Na_2_WO_4_ in the precursor sol was 7.0 mmol, *R* of the membrane became almost one-tenth of that of standard powder data. This further proved that the amount of Na_2_WO_4_ in the precursor sol significantly affected the c-plane orientation of the synthesized h-WO_3_ membranes. In total, 3.5 to 5.0 mmol of Na_2_WO_4_ was found to be effective to prepare h-WO_3_ membranes with high c-plane orientation.

**Figure 13:**
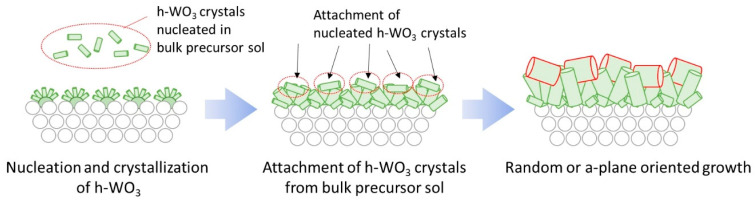
Illustration of the anticipated effect of bulk precursor sol nucleation on crystal orientation of membrane.

The reason why the amount of Na_2_WO_4_ in precursor sol affected the c-plane orientation of the membranes may be explained as follows. When the amount of Na_2_WO_4_ in the precursor sol was below 5.0 mmol, nucleation and crystallization of h-WO_3_ are expected to be mainly occurring near the seeds embedded on the tubular substrate surface. Numerous nuclei must have selectively grown vertically to the substrate surface due to the neighboring nuclei which limits the growth parallel to the substrate surface, as discussed in Figure 9 of Section 3.2. In contrast, when the amount of Na_2_WO_4_ in the precursor sol exceeded 6.0 mmol, nucleation and crystallization of the h-WO_3_ may have taken place primarily in the bulk precursor sol due to the high tungsten concentration. The h-WO_3_ crystals nucleated in the bulk precursor sol shall attach on the substrate surface and become incorporated into the h-WO_3_ membrane starting to grow on the surface of the tubular substrate as shown in Figure 13, thus decreasing the degree of c-plane orientation of the membrane. Furthermore, the number of nuclei generated in the bulk precursor sol is likely to increase as the precursor sol reaches closer to saturation and this must have led to attachment of more h-WO_3_ crystals from the bulk precursor sol onto the substrate surface. A precursor sol containing a moderate amount of Na_2_WO_4_, which is large enough to grow and densify the membrane but low enough to prevent vast nucleation and crystallization of h-WO_3_ in the bulk precursor sol, seems to be the key for realizing a h-WO_3_ membrane satisfying both high densification and high c-plane orientation.

### 3.4. PV Performance

Table 1 shows the PV performance of the h-WO_3_ membrane obtained from precursor sols with various amounts of Na_2_WO_4_. The membrane performance drastically deteriorated when the amount of Na_2_WO_4_ exceeded 6.0 mmol. The total flux of membranes synthesized with precursor sols with 3.5 and 5.0 mmol of Na_2_WO_4_ approximately doubled compared to those prepared with 6.0 and 7.0 mmol of Na_2_WO_4_. The separation factor also drastically changed between 5.0 and 6.0 mmol of Na_2_WO_4_. This seemed to be closely related to the change in the degree of c-plane orientation of the membranes shown in Figure 12. The membranes showing high performance exhibited an obviously high degree of c-plane orientation. Therefore, the crystal orientation must have highly affected the membrane performance. To understand this reason, the permeance of water and CH_3_COOH were compared. The fluctuation in CH_3_COOH permeance was rather small regardless of the change in total flux while the change in water permeance drastically increased along with the increase in total flux. This means that the pathway for only water molecules increased. As we have revealed in our previous study, the separation mechanism of h-WO_3_ membranes is based on molecular sieving [26]. Because the pores of h-WO_3_ is running along the c-axis of h-WO_3_ crystals and the water-permeable pores are smaller than CH_3_COOH molecules, the higher c-plane orientation must have resulted in more pathways for water molecules without increasing those for CH_3_COOH molecules. The growth of non-c-plane oriented h-WO_3_ crystals attached and incorporated in the membrane must be prevented to realize high membrane performance.

**Figure 14:**
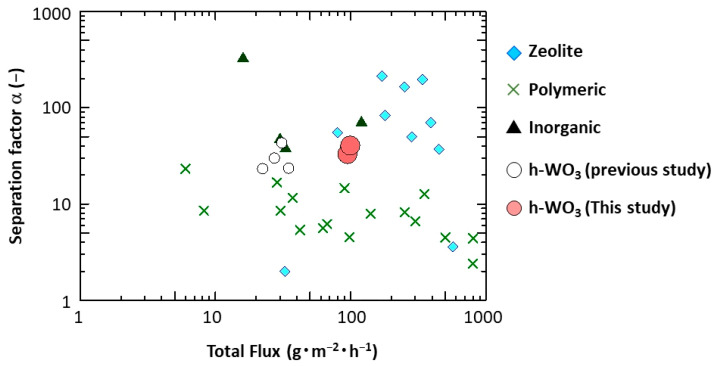
Performance of highly c-plane oriented h-WO_3_ membrane compared to our previous h-WO_3_ membranes and conventional membranes. (The open circles indicate the h-WO_3_ membranes reported in our previous study, reference [26]; the cross marks indicate performances of conventional polymeric membranes reported in references [29,30,31]; the triangle marks indicate performances of conventional inorganic membranes reported in references [32,33]; the diamond marks indicate the conventional zeolite membranes reported in reference [9,12,14,20,34,35].)

Figure 14 shows the PV performance for separation of 90:10 wt % CH_3_COOH/water mixture of the highly c-plane oriented h-WO_3_ membranes obtained in this study (prepared by precursor sols containing 3.5 and 5.0 mmol of Na_2_WO_4_) compared to our previous study and membranes comprised of conventional materials. Compared to our previous report, the total flux was drastically enhanced in this study. Indeed, the total flux was increased by more than twice of that of the previous study. Moreover, the performances of the developed membranes seem to be higher than conventional polymeric membranes [29,30,31] and are becoming closer to those of conventional inorganic membranes [9,12,14,20,32,33,34,35], including zeolite membranes [9,12,14,20,34,35], which are expected to be promising candidate membranes for separating CH_3_COOH/water mixtures.

**Figure 15:**
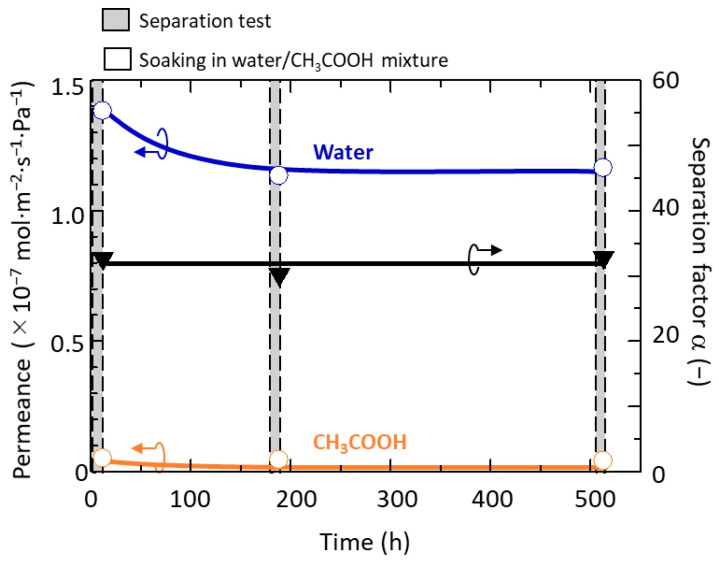
Performance of highly c-plane oriented h-WO_3_ membrane during long-term-separation test.

The stability of the h-WO_3_ membrane was evaluated by a long-term-separation test using the membrane synthesized with 3.5 mmol of Na_2_WO_4_. The PV performance of the membrane was repeatedly evaluated before and after storing in a 90:10 wt % CH_3_COOH/water mixture with a pH below 0 for a particular period at room temperature. The result is shown in Figure 15. Even after repeated testing for more than 500 h, the separation factor was maintained almost constant. Both water and CH_3_COOH permeance decreased by about 20% from the first test to the second test; however, the permeance was still high compared to our previous report [26] and maintained nearly constant thereafter for more than 300 h. This indicated that the h-WO_3_ membrane is durable against acidic solvents and that its performance can be maintained even during a long-term-separation test.

So far, several zeolite membranes have been evaluated using long-term separation tests [10,11,12,13,15,17]. The initial decrease in permeability has been observed in almost all cases. Masuda et al. anticipated that this is due to the plugging by partial insertion of the CH_3_COOH molecule into the zeolite pores [13]. Thus, a similar phenomenon may have taken place in this study. However, compared with a conventional study tested in a similar testing condition of 90:10 wt % CH_3_COOH/water mixture at 348 K, the permeability of MOR-type zeolite membrane decreased to nearly half after 30 h of PV test [11] while the developed h-WO_3_ membrane decreased by only 20% and stabilized. This implies that the plugging of h-WO_3_ pores caused by the CH_3_COOH molecule was much less compared to the mordenite pores. From this result, there is a possibility that the h-WO_3_ membrane has a better fouling resistance compared to the conventionally reported membrane.

## 4. Conclusions

In this work, the effect of synthesis conditions of h-WO_3_ membranes with c-plane orientation on tubular substrates and their CH_3_COOH/water separation performance were investigated. The h-WO_3_ membranes were prepared by hydrothermal synthesis using a precursor sol containing various amounts of Na_2_WO_4_ in the presence of tubular substrates with seeds embedded on only their outside surface. The method to embed the amorphous h-WO_3_ seeds selectively on the outer surface of the tubular substrates was found to play an important role against densification and c-plane orientation of the h-WO_3_ membranes. In addition, the amount of Na_2_WO_4_ in the precursor sol significantly affected crystal orientation of the membrane. A precursor sol with 3.5 and 5.0 mmol of Na_2_WO_4_ was effective to simultaneously satisfy c-plane orientation and densification of the membrane while an excess amount of Na_2_WO_4_ drastically decreased the degree of c-plane orientation. A highly c-plane oriented h-WO_3_ membrane was successfully obtained under the optimized condition, which had a c-plane orientation index of approximately 10 and exhibited a maximum separation factor of 40.0 with a water permeance as high as 1.53 × 10^−7^ mol·m^−2^·s^−1^·Pa^−1^ in a 90:10 wt % CH_3_COOH/water mixture. This corresponded to a greater than five-times higher c-plane orientation index and a greater than two-times higher water permeance compared to our previous study. Furthermore, it was found that the CH_3_COOH/water separation ability of the highly c-plane oriented h-WO_3_ membrane can be maintained while being stored in 90:10 wt % CH_3_COOH/water mixture with pH < 0 for 500 h, proving its high acidic resistance and stability of performance in an acidic environment. The highly c-plane oriented h-WO_3_ membrane seems to be a possible candidate for separation of water from acidic solutions.

## Figures and Tables

**Table 1:** PV performance of h-WO_3_ membranes synthesized using precursor sols containing different amounts of Na_2_WO_4_ for separation of CH_3_COOH/water mixtures.

Membrane No.	Amount of Na_2_WO_4_ in Precursor Sol	CH_3_COOH/Water in Feed	T	α	Total Flux	Permeance (mol∙m^−2^∙s^−1^∙Pa^−1^)	
	(mmol)	(wt %:wt %)	(K)	(-)	(g∙m^−2^∙h^−1^)	Water	CH_3_COOH
M1	3.5	90:10	353	32.7	95.4	1.38 × 10^−7^	4.99 × 10^−9^
M2	5.0	90:10	353	40.0	100.0	1.53 × 10^−7^	4.48 × 10^−9^
M3	6.0	90:10	353	8.0	52.5	5.13 × 10^−8^	6.93 × 10^−9^
M4	7.0	90:10	353	12.3	43.5	5.20 × 10^−8^	4.45 × 10^−9^

## Data Availability

Data is contained within the article or supplementary material.

## Supplementary Materials

The following are available online at https://www.mdpi.com/2077-0375/11/1/38/s1, Figure S1: Difference in c-plane orientation of h-WO_3_ membranes prepared on (a) flat-type substrate and (b) tubular substrate under the same preparation condition in previous study.

## References

[1] Mahdi T., Ahmad A., Nasef M.M., Ripin A. (2016). State-of-the-Art Technologies for Separation of Azeotropic Mixtures. Sep. Purif. Rev..

[2] Wee S.L., Tye C.T., Bhatia S. (2008). Membrane separation process—Pervaporation through zeolite membrane. Sep. Purif. Technol..

[3] Jullok N., Luis P., Degrève J., Bruggen B.V. (2014). Cascaded pervaporation process for dehydration of acetic acid. Chem. Eng. Sci..

[4] Huang Y., Baker R.W., Vane L.M. (2010). Low-energy distillation-membrane separation process. Ind. Eng. Chem. Res..

[5] Kunnakorn D., Rirksomboon T., Siemanond K., Aungkavattana P., Kuanchertchoo N., Chuntanalerg P., Hemra K., Kulprathipanja S., James R.B., Wongkasemjit S. (2013). Techno-economic comparison of energy usage between azeotropic distillation and hybrid system for water-ethanol separation. Renew. Energ..

[6] Sholl D.S., Lively R.P. (2016). Seven chemical separations to change the world. Nature.

[7] Ray S.K., Sawant S.B., Joshi J.B., Pangarkar V.G. (1998). Dehydration of acetic acid by pervaporation. J. Membr. Sci..

[8] Matsukata M. (2010). Jouryuu–mukimaku hiburido purosesu niyoru enerugi sakugen no kanousei. Petrotech.

[9] Zhang Y., Qiu X., Hong Z., Du P., Song Q., Gu X. (2019). All-silica DD3R zeolite membrane with hydrophilic-functionalized surface for efficient and highly-stable pervaporation dehydration of acetic acid. J. Membr. Sci..

[10] Li Y., Zhu M., Hu N., Zhang F., Wu T., Chen X., Kita H. (2018). Scale-up of high performance mordenite membranes for dehydration of water-acetic acid mixtures. J. Membr. Sci..

[11] Zhu M.H., Xia S.L., Hua X.M., Feng Z.J., Hu N., Zhang F., Kumakiri I., Lu Z.H., Chen X.S., Kita H. (2014). Rapid preparation of acid-stable and high dehydration performance mordenite membranes. Ind. Eng. Chem. Res..

[12] Li G., Kikuchi E., Matsukata M. (2003). Separation of water-acetic acid mixtures by pervaporation using a thin mordenite membrane. Sep. Purif. Technol..

[13] Masuda T., Otani S., Tsuji T., Kitamura M., Mukai S.R. (2003). Preparation of hydrophilic and acid-proof silicalite-1 zeolite membrane and its application to selective separation of water from water solutions of concentrated acetic acid by pervaporation. Sep. Purif. Technol..

[14] Li G., Kikuchi E., Matsukata M. (2003). A study on the pervaporation of water–Acetic acid mixtures through ZSM-5 zeolite membranes. J. Membr. Sci..

[15] Sano T., Ejiri S., Yamada K., Kawakami Y., Yanagishita H. (1997). Separation of acetic acid-water mixtures by pervaporation through silicalite membrane. J. Membr. Sci..

[16] Nagase T., Kiyozumi Y., Hasegawa Y., Inoue T., Ikeda T., Fujio M. (2007). Dehydration of Concentrated Acetic Acid Solutions by Pervaporation Using Novel MER Zeolite Membranes. Chem. Lett..

[17] Yamanaka N., Itakura M., Kiyozumi Y., Ide Y., Sadakane M., Sano T. (2012). Acid stability evaluation of CHA-type zeolites synthesized by interzeolite conversion of FAU-type zeolite and their membrane application for dehydration of acetic acid aqueous solution. Microporous Mesoporous Mater..

[18] Yamanaka N., Itakura M., Kiyozumi Y., Ide Y., Sadakane M., Sano T. (2013). Effect of Structure-Directing Agents on FAU–CHA Interzeolite Conversion and Preparation of High Pervaporation Performance CHA Zeolite Membranes for the Dehydration of Acetic Acid Solution. Bull. Chem. Soc. Jpn..

[19] Yajima K., Hagio T., Miyahara M., Takahashi N., Niino M., Isomura M., Yoshida S. (2014). Development of DDR-type Zeolite Membrane on Multichanneled Support Having Large Membrane Area. Zeolite.

[20] Zhang Y., Chen S., Shi R., Du P., Qiu X., Gu X. (2018). Pervaporation dehydration of acetic acid through hollow fiber supported DD3R zeolite membrane. Sep. Purif. Technol..

[21] Miao B., Zeng W., Hussain S., Mei Q., Xu S., Zhang H., Li Y., Li T. (2015). Large scale hydrothermal synthesis of monodisperse hexagonal WO_3_ nanowire and the growth mechanism. Mater. Lett..

[22] Jiao Z., Wang J., Ke L., Sun X.W., Demir H.V. (2011). Morphology-tailored synthesis of tungsten trioxide (hydrate) thin films and their photocatalytic properties. ACS Appl. Mater. Interfaces.

[23] Sun W., Yeung M.T., Lech A.T., Lin C.W., Lee C., Li T., Duan X., Zhou J., Kaner R.B. (2015). High surface area tunnels in hexagonal WO_3_. Nano Lett..

[24] Bowen T.C., Noble R.D., Falconer J.L. (2004). Fundamentals and applications of pervaporation through zeolite membranes. J. Membr. Sci..

[25] Takacs M., Ducso C., Pap A.E. (2015). Fine-tuning of gas sensitivity by modification of nano-crystalline WO_3_ layer morphology. Sens. Actuators B Chem..

[26] Kunishi H., Hagio T., Wada S., Kamimoto Y., Ichino R. (2020). Development of novel nanoporous hexagonal tungsten oxide membrane for separation of water/acetic acid mixtures via pervaporation. J. Membr. Sci..

[27] Zhang X., Liu H., Wang A., Wang J. (2007). Factors affecting the formation of zeolite seed layers and the effects of seed layers on the growth of zeolite silicalite-1 membranes. Front. Chem. Eng. China.

[28] Zhang X., Liu H., Yeung K.L. (2006). Influence of seed size on the formation and microstructure of zeolite silicalite-1 membranes by seeded growth. Mater. Chem. Phys..

[29] Aminabhavi T.M., Toti U.S. (2003). Pervaporation separation of water–acetic acid mixtures using polymeric membranes. Des. Monomers. Polym..

[30] Aminabhavi T.M., Naik H.G. (2002). Synthesis of Graft Copolymeric Membranes of Poly(vinyl alcohol) and Polyacrylamide for the Pervaporation Separation of Water/Acetic Acid Mixtures. J. Appl. Polym. Sci..

[31] Jullok N., Darvishmanesh S., Luis P., Bruggen B.V. (2011). The potential of pervaporation for separation of acetic acid and water mixtures using polyphenylsulfone membranes. Chem. Eng. J..

[32] Tanaka S., Yasuda T., Katayama Y., Miyake Y. (2011). Pervaporation dehydration performance of microporous carbon membranes prepared from resorcinol/formaldehyde polymer. J. Membr. Sci..

[33] Sommer S., Melin T. (2005). Performance evaluation of microporous inorganic membranes in the dehydration of industrial solvents. Chem. Eng. Process..

[34] Zhang Y., Nakasaka Y., Tago T., Hirata A., Sato Y., Masuda T. (2015). Preparation and optimization of mordenite nanocrystal-layered membrane for dehydration by pervaporation. Microporous Mesoporous Mater..

[35] Zhu M.H., Kumakiri I., Tanaka K., Kita H. (2013). Dehydration of acetic acid and esterification product by acid-stable ZSM-5 membrane. Microporous Mesoporous Mater..

