# Supplementary material for: Synthesis of C-Plane Oriented Hexagonal Tungsten Oxide Membranes on Tubular Substrates and Their Acetic Acid/Water Separation Performances
Source: Membranes (Basel). 2021 Jan 5;11(1):38. doi: 10.3390/membranes11010038 (PMC7824756; doi:10.3390/membranes11010038)
Supplement: Supplementary file 1 [file membranes-11-00038-s001.pdf]

Supplementary Material

Synthesis of C-Plane Oriented Hexagonal Tungsten Oxide Membranes on Tubular Substrates and Their Acetic Acid/Water Separation Performances

Hiroto Kunishi [1], Shintaro Wada [2], Yuki Kamimoto [1,3], Ryoichi Ichino [1,3], Yan Lin [4], Long Kong [4], Liang Li [4] and Takeshi Hagio [1,3,*]

1 Department of Chemical Systems Engineering, Graduate School of Engineering, Nagoya University, Furo-cho, Chikusa-ku, Nagoya, Aichi 464-8603, Japan; kunishi.hiroto@d.mbox.nagoya-u.ac.jp (H.K.); yuki.kamimoto@mirai.nagoya-u.ac.jp (Y.K.); ichino.ryoichi@material.nagoya-u.ac.jp (R.I.)

2 Department of Materials Science and Engineering, School of Engineering, Nagoya University, Furo-cho, Chikusa-ku, Nagoya, Aichi 464-8603, Japan; wada.shintaro@d.mbox.nagoya-u.ac.jp

3 Institute of Materials Innovation, Institutes of Innovation for Future Society, Nagoya University, Furo-cho, Chikusa-ku, Nagoya, Aichi 464-8601, Japan

4 School of Environmental Science and Engineering, Shanghai Jiao Tong University, 800 Dongchuan Road, Shanghai 200240, China; linyan2002@sjtu.edu.cn (Y.L.); longmao88@sjtu.edu.cn (L.K.); liangli117@sjtu.edu.cn (L.L.)

* Correspondence: hagio@mirai.nagoya-u.ac.jp; Tel.: +81-52-747-6594

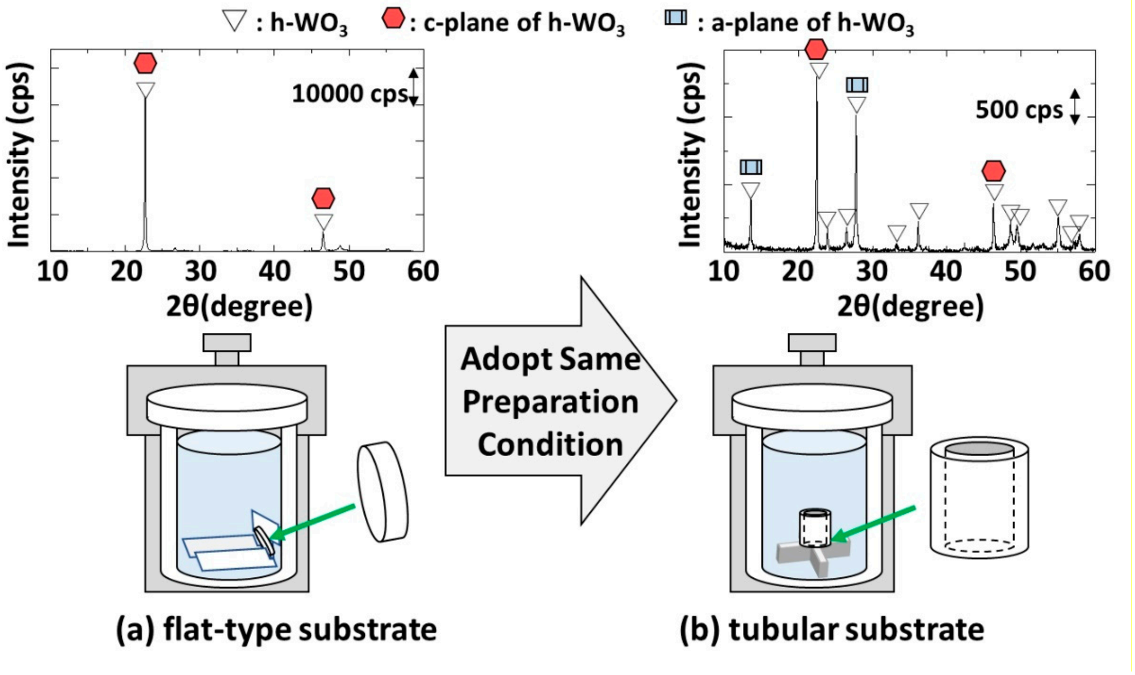

Figure 1. Difference in c-plane orientation of h-WO3 membranes prepared on (**a**) flat-type substrate and (**b**) tubular substrate under the same preparation condition in previous study.
